# A systematic analysis of contemporary whole exome sequencing capture kits to optimise high-coverage capture of CCDS regions

**DOI:** 10.1093/nargab/lqaf115

**Published:** 2025-09-01

**Authors:** Fernando Vázquez López, James J Ashton, Guo Cheng, Sarah Ennis

**Affiliations:** Department of Human Genetics and Genomic Medicine, University of Southampton, Southampton, SO16 6YD, UK; Department of Human Genetics and Genomic Medicine, University of Southampton, Southampton, SO16 6YD, UK; Department of Paediatric Gastroenterology, Southampton Children’s Hospital, Southampton, SO16 6YD, UK; Department of Human Genetics and Genomic Medicine, University of Southampton, Southampton, SO16 6YD, UK; NIHR Southampton Biomedical Research Centre, University Hospital Southampton, Southampton, SO16 6YD, UK; Department of Human Genetics and Genomic Medicine, University of Southampton, Southampton, SO16 6YD, UK

## Abstract

Whole exome sequencing (WES) is a well-established tool for clinical diagnostics, is more cost-effective and faster to analyse than whole genome sequencing and has been implemented to uplift diagnostic rates in human disease. However, challenges remain to achieve comprehensive and uniform coverage of targets, and high sensitivity and specificity. Differences in genomic target regions and exome capture mechanism between kits may lead to differences in overall coverage uniformity and capture efficiency. Here, we analyse the efficiency of a range of off-the-shelf exome sequencing (ES) kits in capturing their reported targets and the consensus coding sequence (CCDS) regions. Our results show Twist Custom Exome, Twist Human Comprehensive Exome, and Roche KAPA HyperExome V1 perform particularly well at capturing their target regions at 10X and 20X coverage and achieve the highest capture efficiency of CCDS regions upon read downsampling. This was the case despite both Twist kits targeting less than 37Mb in the genome. Our analysis highlights the impact of kit target design on capture efficiency in WES, with kit target size and uniformity of coverage impacting the capture efficiency of CCDS regions. This benchmark will help researchers to make an informed decision based on their needs.

## Introduction

Whole exome sequencing (WES) is a well-established tool for Mendelian disease gene discovery [1]. While the exome comprises 1–2% of the human genome, it has been reported to contain ∼85% of all described disease-causing variants [2]. This makes WES a more cost-effective technique than whole genome sequencing (WGS) for diagnostics, and it can deliver higher coverage to detect rare variants [3]. WGS provides even and unbiased coverage of coding regions (especially GC-rich regions), generates more accurate variant calls, and due to its gapless nature enables more accurate detection of structural variants (SVs) [4]. However, due to lower sequencing costs and experimental evidence of diagnostic yield in copy number variation (CNV) detection, WES has been proposed as a first-tier diagnostic test [5, 6]. Furthermore, the lower volume of data generated reduces storage costs and processing time. This technique has been successfully implemented to uplift diagnostic rates for a range of genetic disorders [7, 8], with evidence for its utility in variant discovery and detection in Mendelian and non-Mendelian conditions [9, 10].

Hybrid capture is the main technique used for WES. A capture kit consists of bespoke molecular probes (biotinylated DNA or RNA oligonucleotides) that are complementary to the target regions. After hybridisation and target capture with beads, non-target sequences are washed away, so that the enriched sample can be eluted and processed for DNA sequencing. Hybrid capture can be conducted via reactions in solution or reactions on a solid support [11]. Some of the main challenges in the design and development of whole exome capture technologies are achieving comprehensive and uniform target coverage, and high sensitivity and specificity. Major sources of coverage bias in WES are extreme GC allele content and mappability issues such as repeat elements and segmental duplications [4, 12]. Coverage bias can have a large effect on the robustness of single nucleotide variant (SNV) and CNV calling from exome sequencing (ES) data [13]. There is a wide range of whole exome capture chemistries in the market. These differ in their target genomic regions, sequence features, probe length and capture mechanism. These differences may impact overall coverage uniformity and capture efficiency of specific targets, and therefore variant calling sensitivity and accuracy. Furthermore, a high average coverage depth does not guarantee high enough coverage for individual targets [14, 15].

The consensus coding sequence (CCDS) project aims to identify a core set of human protein coding regions that are consistently annotated and of high quality [16]. CCDS regions are highly curated and address inconsistencies between RefSeq annotation [17] from the National Center for Biotechnology Information (NCBI) and Ensembl annotation [18] from The European Molecular Biology Laboratory's European Bioinformatics Institute (EMBL-EBI). Here, we report on the capture efficiency of a range of WES capture kits from different manufacturers and assess this against their intended targets and CCDS regions. These kits were selected to include the most relevant chemistries currently in use, and samples were retrieved from previous published research, research collaborations, and in-house sequencing. Table 1 gives on overview of the capture kits analysed. A high and uniform coverage of these exonic regions will help to uplift diagnostic rates, and this benchmark will help researchers to make an informed decision based on their needs.

**Table 1.** Coverage of CCDS regions by the intended targets of each WES capture kit. Coverage was calculated as the intersection between the genomic regions defined in the BED file of each capture kit and CCDS regions with and without padding. Genomic size of CCDS regions: 33 153 625 base pairs. Genomic size of CCDS regions ± 25 bp padding: 42 771 721 base pairs

Whole Exome Capture Kit	Manufacturer	Target size (bp)	Coverage of CCDS	Coverage of CCDS ± 25 bp padding
Custom Exome Capture	Twist Biosciences	34 883 866	0.9943	0.7717
DNA Prep with Exome 2.5 Enrichment	Illumina	37 453 133	0.9949	0.7813
Easy Exome Capture V5	MGI	69 335 731	0.996	0.8741
ExomeMax V2	MedGenome	62 436 679	0.9951	0.9061
Human Comprehensive Exome	Twist Biosciences	36 510 191	0.9991	0.7783
KAPA HyperExome V1	Roche	42 988 611	0.9786	0.8734
SureSelect Human All Exon V5	Agilent	50 446 305	0.885	0.8387
SureSelect Human All Exon V6	Agilent	60 507 855	0.9178	0.8773
SureSelect Human All Exon V7	Agilent	35 718 732	1	0.7792
SureSelect Human All Exon V8	Agilent	35 131 620	1	0.8214
xGen Exome Hybridisation Panel V1	IDT	38 997 831	0.9871	0.772

## Materials and methods

### Sample preparation and sequencing

For all capture kits, 10 representative samples were randomly selected for analysis. All of these samples were sequenced on paired-end sequencing. Samples from the Illumina DNA Prep with Exome 2.5 Enrichment, Roche KAPA HyperExome V1, and Agilent SureSelect Human All Exon V7 and V8 kits were obtained from publicly available datasets. The Twist Custom Capture kit was used to sequence the exome of participants in the SPARC IBD study. Sequencing data from the MGI Easy Exome Capture V5, and MedGenome ExomeMax V2 were shared by the respective points of contact and are not publicly available. Samples from the SureSelect Human All Exon V5 and V6, and Twist Human Comprehensive Exome kits were retrieved from the Southampton IBD study. Access to samples from IDT xGen Exome Hybridisation Panel V1 was granted under an ongoing UK Biobank project. Patients or the public were not involved in the design, conduct, reporting, or dissemination plans of our research. Details on library preparation and sequencing are provided in the research publications and supplementary methods. Details on DNA purification and library preparation for DNA Prep with Exome 2.5 Enrichment samples were not available in the BaseSpace platform. All capture kits analysed are commercially available, except the Twist Custom Capture kit, which is a custom kit designed by the Broad Institute. This kit targets >99% of the regions targeted by Twist Human Core Exome and an extra ∼1.7 Mb. All files were securely transferred to the University of Southampton Iridis 6 High Performance Computing cluster, using WinSCP and the Amazon Web Services client; and passed applicable FastQC (version 0.12.1) metrics for next-generation sequencing. All downstream processing and analysis were conducted in this cluster. Files from all kits were obtained in FASTQ format, except for Twist Custom Capture, in which case data were transferred in the CRAM format.

### Bioinformatic analysis

BED files for the target regions of each capture kit were either obtained from the manufacturer’s website or the point of contact for data sharing. The BED file for the latest release of CCDS regions was downloaded from the UCSC Genome Browser (GRCh38.p14 genome assembly) (release 24–27/10/2022). To analyse the overlap between different capture kit targets and CCDS regions, the intersection of the target regions BED file of each kit with the CCDS BED was calculated. For this, we used bedtools (version 2.31.1) merge and intersect. To ensure uniformity, all raw FASTQ files from sequencing libraries generated by each kit were processed using the same bioinformatic pipeline. This pipeline is based on the Genome Analysis Toolkit (GATK) best practices [19]. Where necessary, FASTQ files are merged to produce one forward- and one reverse-read file. Read alignment is performed using the human reference genome (GRCh38 assembly with Human Leukocyte Antigen (HLA) regions) and Burrow-Wheeler Aligned-Maximal Exact Match (BWA-MEM) (bwa version 0.7.17). After alignment, duplicate reads are marked (Picard MarkDuplicates) and base quality recalibration is conducted (GATK BaseRecalibrator and ApplyBQSR) (jdk version 22.0.1; GATK version 4.6.0.0; Picard version 3.2.0). Downsampling to 40 000 000 reads was conducted using Picard DownsampleSam with default options. This was done for benchmarking purposes, to avoid biases derived from the amount of sequencing output generated for each kit. Furthermore, Trimmomatic (version 0.40) [20], was used to trim the reads of all FASTQ files to a length of 75bp. Reads were trimmed from the end of the reads, and this was followed by alignment and recalibration.

BED files were converted into interval list files using Picard BedToIntervalList, to meet the input requirements of Picard CollectHsMetrics. A padding of ±25 bp was added to the CCDS regions. This is common practice and justified by findings that show a significant amount of disease-causing variation occurring within 25 bp upstream and downstream of splice sites in the genome [21]. A padding of ±25 bp and ±100 bp was added to the kits’ target regions to assess capture efficiency of immediately adjacent intergenic regions. 10% of the exonic variants analysed by Soemedi *et al.* (2017) altered splicing [22]. CollectHsMetrics was applied to assess capture efficiency, coverage depth and uniformity, and other BAM metrics. Picard CollectInsertSizeMetrics was used to estimate library insert size (see Supplementary Methods for details). The Integrative Genomics Viewer (IGV) genome browser (version 2.19.4) was used with the GRCh38 genome assembly.

## Results

### Coverage of CCDS regions by the intended targets of WES capture kits

All kits (except Agilent SureSelect Human All Exon V5 and V6) target > 97.5% of CCDS regions. SureSelect Human All Exon V8, both versions of the Twist kits, and the Illumina DNA Prep with Exome 2.5 Enrichment kit target >99% of CCDS, and each of them targets <38 Mb in the genome (Supplementary Fig. S1). SureSelect Human All Exon V8, both versions of the Twist kits, and the Illumina DNA Prep with Exome 2.5 Enrichment target ∼77–82% of CCDS regions with ± 25 bp padding, whereas capture kits targeting a larger genomic region (MGI Easy Exome Capture V5, Roche KAPA HyperExome V1 and MedGenome ExomeMax V2) target the ±25 bp padded CCDS regions more consistently (>87%) (Supplementary Fig. S2) (Table 1).

### Exome capture efficiency

**Figure 1.**
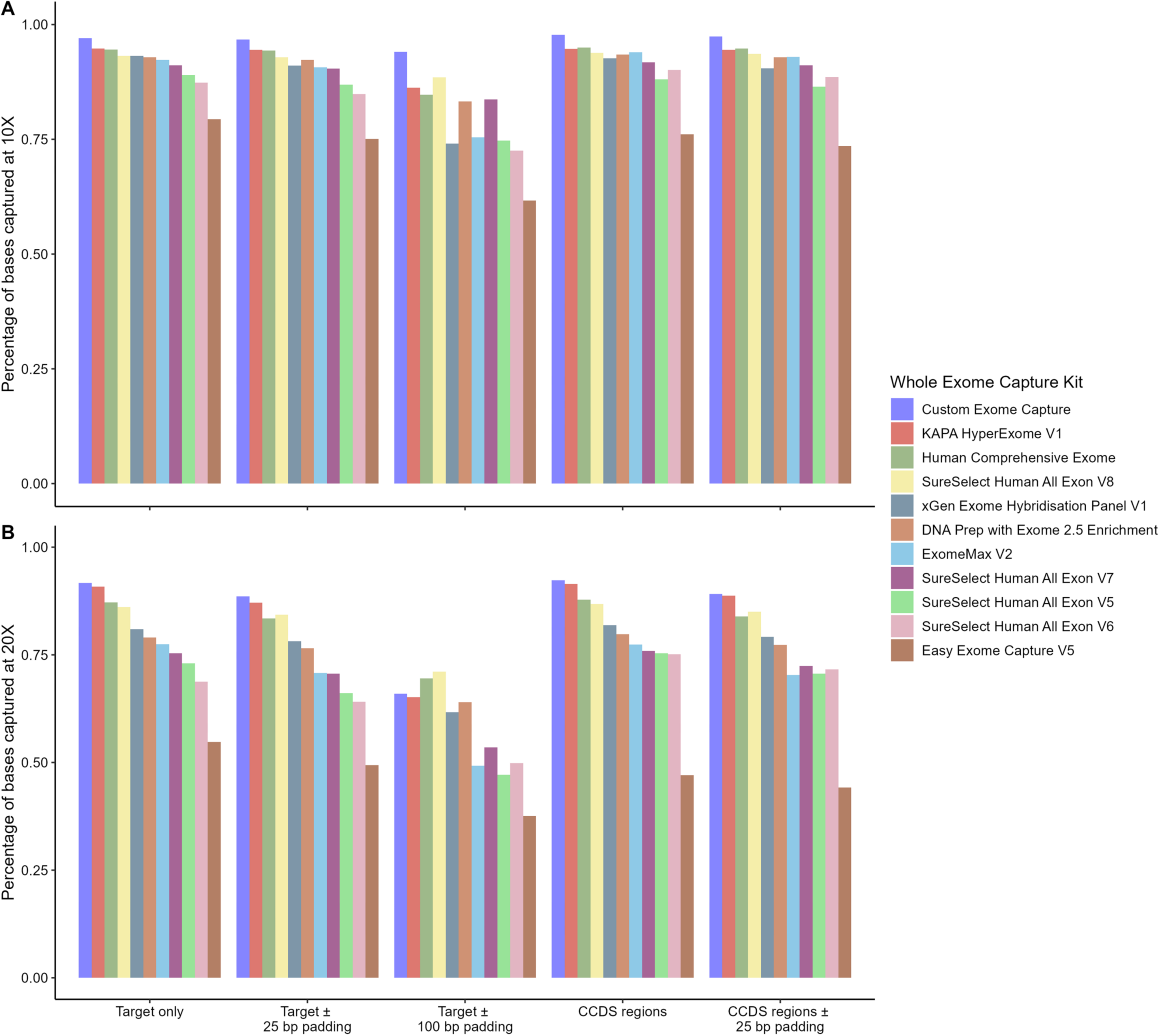
Percentage of target bases or CCDS region bases captured by the probes from each WES capture kit at 10X and 20X coverage. These results are the average across 10 samples for each kit, all of which were downsampled to 40 000 000 reads after alignment. Capture efficiency is measured as the percentage of base pairs covered at a read depth of 10X or 20X by the reads generated using each capture kit. Capture efficiency for the target regions of each kit is provided for no padding, ±25 bp, and ±100 bp padding. Capture efficiency for CCDS regions is provided for no padding and ±25 bp padding.

The results from the WES capture efficiency analysis are shown in Table 2. Twist Custom Exome and Human Comprehensive Exome, and Roche KAPA HyperExome V1 performed particularly well at capturing their target regions at 10X (>94%) (Fig. 1). Easy Exome Capture V5 achieved < 80% capture at 10X. The values for capture efficiency at 10X and 20X with ±25 bp padding were consistent, with the same three kits outperforming competitors, and minor differences overall. Twist Custom Exome achieved a coverage of its targets with ± 100 bp padding at 10X of 94%. Of interest, both Twist Custom Exome and SureSelect Human All Exon V8 had the highest consistency in capture of their targets with ±25 bp and ±100 bp padding at 10X. Twist Human Comprehensive Exome and Roche KAPA HyperExome V1 had the lowest Fold-80 base penalty for their targets, whereas MGI Easy Exome Capture V5 and Agilent SureSelect All Exon V5 and V6 all had values above 2.3 (Table 2). A lower value of this metric is indicative of better coverage uniformity. Twist Custom Exome and Human Comprehensive Exome, and Roche KAPA HyperExome V1 also achieved the highest capture efficiency of CCDS regions at 10X (>94%). All kits analysed showed a small drop in the capture efficiency of the ±25 bp padded regions at 10X. At 20X, both Twist kits and the Roche kit achieved the highest capture of CCDS by at least ∼5%, with Twist Human Comprehensive outperforming Twist Custom (with and without any padding) (Fig. 1). The uniformity of coverage for CCDS regions ±25 bp padding was largely consistent with the results for the intended targets in all kits.

**Table 2.** Whole exome sequencing capture efficiency of the analysed capture kits. These results are the average across ten samples for each kit, all of which were downsampled to 40 000 000 reads. Capture efficiency is measured as the percentage of base pairs covered at a read depth of 10X or 20X by the reads generated using each capture kit. Percentages are provided for the kit target regions only and regions with padding, as well as CCDS regions with and without padding. Coverage depth is the mean read coverage per base pair across the target regions of each capture kit. Coverage uniformity is provided as the Fold-80 Base Penalty, which reports the fold over-coverage (extra sequencing) necessary to raise 80% of target bases to the mean coverage level

Whole Exome Capture Kit	Manufacturer	Target size (Mb)	Coverage depth	Coverage uniformity		Capture at 10X					Capture at 20X						
				Kit target	CCDS ± 25 bp padding	Kit target			CCDS regions		Kit target			CCDS regions			
						Target only	±25 bp padding	±100 bp padding	CCDS	±25 bp padding	Target only	±25 bp padding	±100 bp padding	CCDS	**±25 bp padding**	Sequencer	Source
Custom Exome Capture	Twist Biosciences	34.9	41.74	1.79	1.74	0.970	0.968	0.940	0.978	0.974	0.872	0.835	0.695	0.878	0.839	Illumina NovaSeq6000	SPARC IBD [27, 28]
DNA Prep with Exome 2.5 Enrichment	Illumina	37.5	37.00	1.86	1.88	0.929	0.923	0.832	0.935	0.929	0.810	0.782	0.617	0.819	0.791	Illumina NovaSeq6000	Illumina BaseSpace
Easy Exome Capture V5	MGI	69.3	31.52	3.47	3.20	0.794	0.751	0.617	0.761	0.736	0.548	0.494	0.376	0.471	0.442	MGI DNB-SEQ-G400	Unpublished work
ExomeMax V2	MedGenome	62.4	28.37	1.67	1.64	0.923	0.907	0.754	0.940	0.930	0.730	0.661	0.471	0.754	0.707	Illumina NovaSeq X Plus	MedGenome
Human Comprehensive Exome	Twist Biosciences	36.5	43.47	1.48	1.57	0.945	0.943	0.847	0.950	0.948	0.917	0.886	0.660	0.923	0.891	Illumina NovaSeq X	Southampton IBD study [29]
KAPA HyperExome V1	Roche	43	40.98	1.46	1.55	0.948	0.945	0.862	0.947	0.945	0.909	0.871	0.652	0.915	0.887	Illumina NovaSeq6000	[26]
SureSelect Human All Exon V5	Agilent	50.4	38.10	2.30	2.57	0.890	0.869	0.747	0.881	0.865	0.754	0.706	0.535	0.759	0.724	Illumina HiSeq2000	Southampton IBD study [29]
SureSelect Human All Exon V6	Agilent	60.5	35.25	2.54	2.52	0.873	0.848	0.725	0.901	0.886	0.687	0.641	0.499	0.752	0.716	Illumina HiSeq2000	Southampton IBD study [29]
SureSelect Human All Exon V7	Agilent	35.7	41.71	2.22	2.24	0.911	0.904	0.837	0.918	0.911	0.790	0.765	0.640	0.798	0.773	MGI DNB-SEQ-G400	[15]
SureSelect Human All Exon V8	Agilent	35.1	41.80	1.75	1.78	0.932	0.929	0.885	0.938	0.936	0.861	0.843	0.711	0.868	0.850	MGI DNB-SEQ-G400	[15]
xGen Exome Hybridisation Panel V1	IDT	39	31.38	1.71	1.85	0.932	0.911	0.740	0.927	0.905	0.774	0.708	0.492	0.774	0.703	Illumina NovaSeq6000	UK BioBank [30]

To control for potential bias derived from the different read lengths employed by the kits analysed, capture efficiency was re-assessed after read trimming of FASTQ files, using the shortest length (75bp for xGen Exome Hybridisation Panel V1) as a baseline. The same downsampling to 40 000 000 reads was applied to the aligned BAM files. An overall drop in coverage depth and capture efficiency was apparent after read trimming (Supplementary Table S3). Coverage uniformity as measured by the Fold-80 base penalty also worsened. The Twist Human Comprehensive Exome, Roche KAPA HyperExome V1, and IDT xGen Exome Hybridisation V1 achieved the highest capture efficiency of their intended targets at 10X (>0.93%), which was consistent for a ±25 bp padding. These three kits also outperformed competitors at 20X capture efficiency of their targets. They were the best performing chemistries at consistently capturing ±25 bp padded CCDS regions (>90%), alongside with Agilent SureSelect V8. Supplementary Fig. S3 provides a comparison of the coverage of exon 4 in the *NOD2* gene by one sample from each capture kit, after downsampling of the untrimmed BAMs. The Agilent SureSelect Human All Exon V6 and V8, Roche KAPA HyperExome V1, and Twist Human Comprehensive showed the most uniform coverage of this exon.

## Discussion

In this analysis, we have shown the impact of genomic target size and capture chemistry on capture efficiency in WES, after read downsampling. These differences are apparent in the capture of a kit's own targets and in the capture of CCDS regions. A pattern emerged by which kits that targeted an overall smaller fraction of the genome provided higher depth and efficiency in capturing CCDS, by designing optimally baited chemistries. Furthermore, upon downsampling, kits targeting a larger region of the genome tended to show a lower mean coverage depth of their targets, and poorer capture efficiency at 20X of both their targets and CCDS regions.

The two most recent iterations of Agilent SureSelect Human All Exome (V7 and V8) target a much smaller genomic region than V5 and V6. Nevertheless, they fully target CCDS, but this drops to 77–82% with ±25 bp padding. However, in our analysis, both Twist Custom Exome Capture and Human Comprehensive Exome, and Roche KAPA HyperExome V1 achieved the best capture of their own targets and CCDS, at 10X and 20X coverage. They showed consistent capture of CCDS regions ±25 bp padding even at 20X coverage. Due to their small target size they may be cost effective options for the efficient capture of CCDS regions (especially both Twist kits). High coverage uniformity of targets correlates with high capture efficiency in the Twist and Roche kits. Conversely, MGI Easy Exome Capture V5 and Agilent SureSelect Human All Exon V5 and V6 had both poor uniformity of coverage of their targets, and poor capture efficiency. A similar pattern was apparent in the uniformity of coverage and capture efficiency of CCDS regions ±25 bp padding.

These results are consistent with the analysis conducted by Belova *et al.* (2022), which showed that Agilent SureSelect Human All Exome V8 achieved higher capture efficiency of its target regions that V7 [15]. Belova *et al.* (2025) also found that Agilent SureSelect V8 and Roche KAPA HyperExome perform well at capturing their own target regions at 10X and 20X [23]. Capture efficiency was ∼96–98% after downsampling to 50 000 000 reads. Upon normalising for read counts, Zhou *et al.* (2021) obtained very similar capture efficiency values at 10X and 20X for Agilent SureSelect V7 and IDT xGen Exome Panel V1, as well as for a Twist kit (Human Core Exome) [14]. All values were >95%.

We conducted read downsampling and used the same bioinformatic pipeline for all capture kits to remove bias derived from the capture chemistry and sequencing process. Despite this, differences in read length and overall size of genomic target regions may still contribute to bias in the mean depth of coverage and capture efficiency. Furthermore, the different library preparation and sequencing protocols used may be introducing batch effects into the data, which could affect the quality of the mapping downstream. However, due to the nature of this analysis this is an unavoidable sequela, as these differences cannot be fully controlled for. Previous analysis by Sun *et al.* (2024) showed that the choice of bioinformatic analysis pipeline has a greater impact on the output of WES than the sequencing platform and library preparation [24], which highlights the utility of the *in-silico* normalisation steps taken in this analysis. Read trimming to 75 bp was implemented to control for differences in sequencing read length. After this step, both the Twist Human Comprehensive Exome and KAPA HyperExome V1 kits remained among the top three for capture efficiency of their targets and CCDS regions. While Twist Custom capture fell further behind, the IDT xGen Exome Hybridisation Panel V1 kit, which did not undergo a drop in performance (as its read length was the baseline for trimming), was now among the top three kits. It is worth highlighting the differential impact of read trimming on the performance of kits with the same original read length. This is the case of both Twist kits, which employ 150 bp reads. Furthermore, despite undergoing the harshest read trimming, both Twist Human Comprehensive and KAPA HyperExome V1 (150 bp reads) only showed small drops in capture efficiency.

The three kits demonstrating the highest capture efficiency of their targets and CCDS regions also have the highest mean coverage depth after read downsampling (>40X). However, this alone does not explain variation in performance, as shown by the fact that MedGenome ExomeMax V2 has better capture efficiency of its target and CCDS regions than Agilent SureSelect Human All Exome V5 and V6, despite having lower mean coverage depth. The better uniformity of coverage displayed by ExomeMax V2 is likely to be contributing to its capture efficiency. This was replicated after read trimming, with the three best performing kits also having the highest mean coverage depth (>30X). Furthermore, while read downsampling helped to minimise mean coverage depth bias, it also had a larger impact on the capture of target and CCDS regions at 20X than at 10X, especially for kits with a larger genomic target size (Illumina DNA Prep, MGI Easy Exome Capture V5, MedGenome ExomeMax V2, Agilent SureSelect Human All Exon V5 and V6, and IDT xGen Exome Hybridisation Panel V1) (Supplementary Table S2). The percentage of unique reads varied between ∼68–95%, indicating another potential source of bias (Supplementary Table S1). The high rate of read duplication in the Twist Custom Capture samples may have been caused by suboptimal DNA concentrations prior to PCR or issues during library preparation.

Our analysis appears to reveal a trade-off between WES capture chemistries targeting a smaller genomic region, which display high mean coverage depth and capture efficiency of CCDS regions, and kits targeting a larger genomic region. The latter display a lower mean coverage depth after read downsampling but are still enhanced to capture intergenic regions (CCDS regions ±25 bp padding). Ultimately, the choice of capture kit for WES will depend on the researchers’ specific needs. As WES capture kits have evolved, there has been an attempt to merge towards a more common exome. As data sharing becomes ever more critical for ultra-rare disease diagnostics and data-rich AI-based research, science and the research community will be better served by greater commonality and standardisation of omic data outputs. This is demonstrated by projects aiming to establish a highly validated consensus set of exonic sequences in the human genome, such as CCDS, RefSeq and GENCODE (Ensembl). Finally, this analysis could be enhanced by studying other metrics such as GC content [14], and expanding the capture efficiency analysis to GENCODE gene annotations [23], which cover a larger region of the genome than CCDS. Where necessary, the diagnostic yields of a selection of capture kits could be evaluated against clinically relevant mutations in HGMD [25].

## Supplementary Material

lqaf115_Supplemental_Files

## Data Availability

Where stated, raw sequencing data is publicly available. Otherwise, data cannot be publicly shared due to data sharing restrictions. The BioProject accession for Liang *et al.* (2022) is PRJNA824211 [26], and the accession for Belova *et al.* (2022) is PRJNA832525 [15]. The Illumina DNA Prep with Exome 2.5 Enrichment data were obtained from BaseSpace, with project ID NA12878. The SPARC IBD data are available upon approved application to Crohn's & Colitis Foundation IBD Plexus (https://www.crohnscolitisfoundation.org/research/plexus). Data from the Southampton Genetics of Inflammatory Bowel Disease study can be shared via appropriate data sharing arrangements.
