# Supplementary material for: A systematic analysis of contemporary whole exome sequencing capture kits to optimise high-coverage capture of CCDS regions
Source: NAR Genom Bioinform. 2025 Sep 1;7(3):lqaf115. doi: 10.1093/nargab/lqaf115 (PMC12408908; doi:10.1093/nargab/lqaf115)
Supplement: lqaf115_Supplemental_Files [file lqaf115_supplemental_files.zip › Supplementary_Figures_20062025.docx]

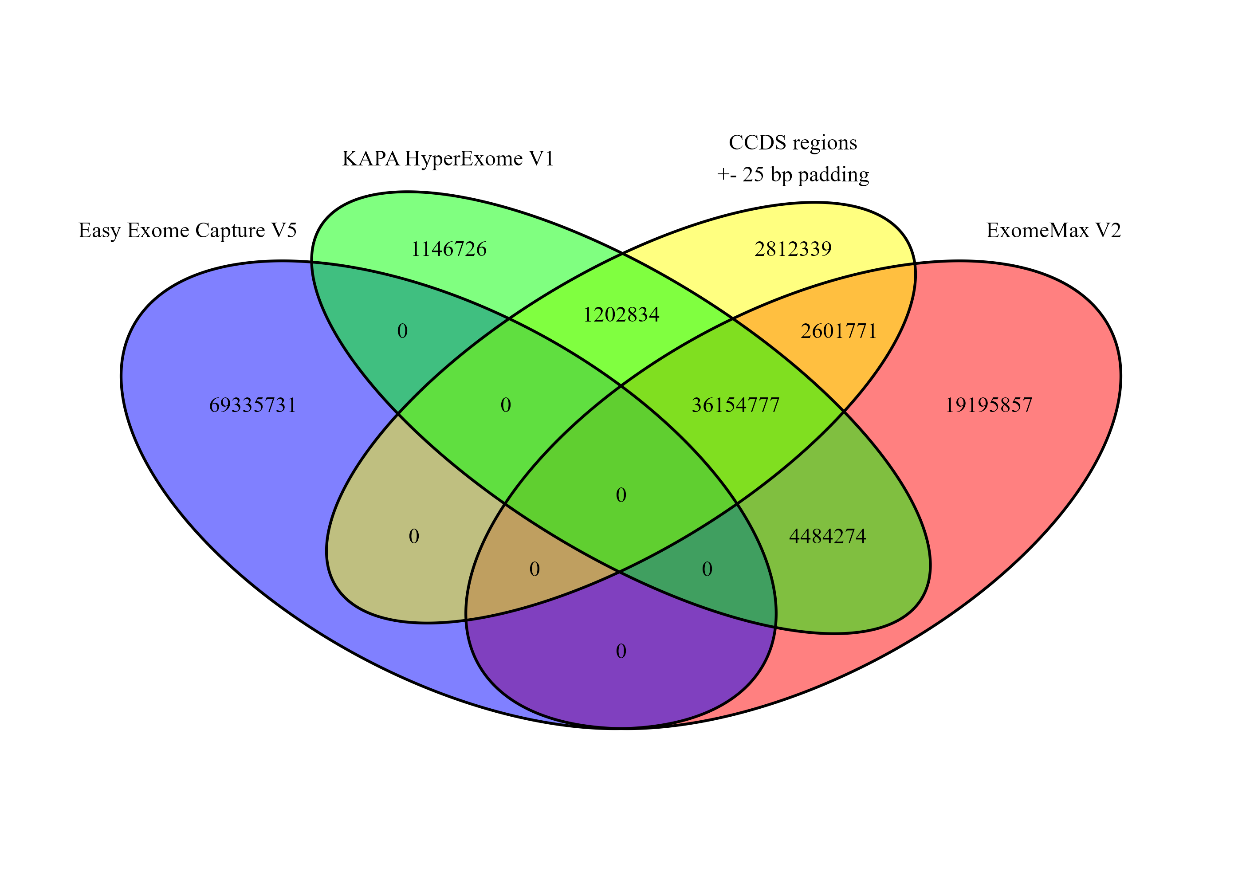

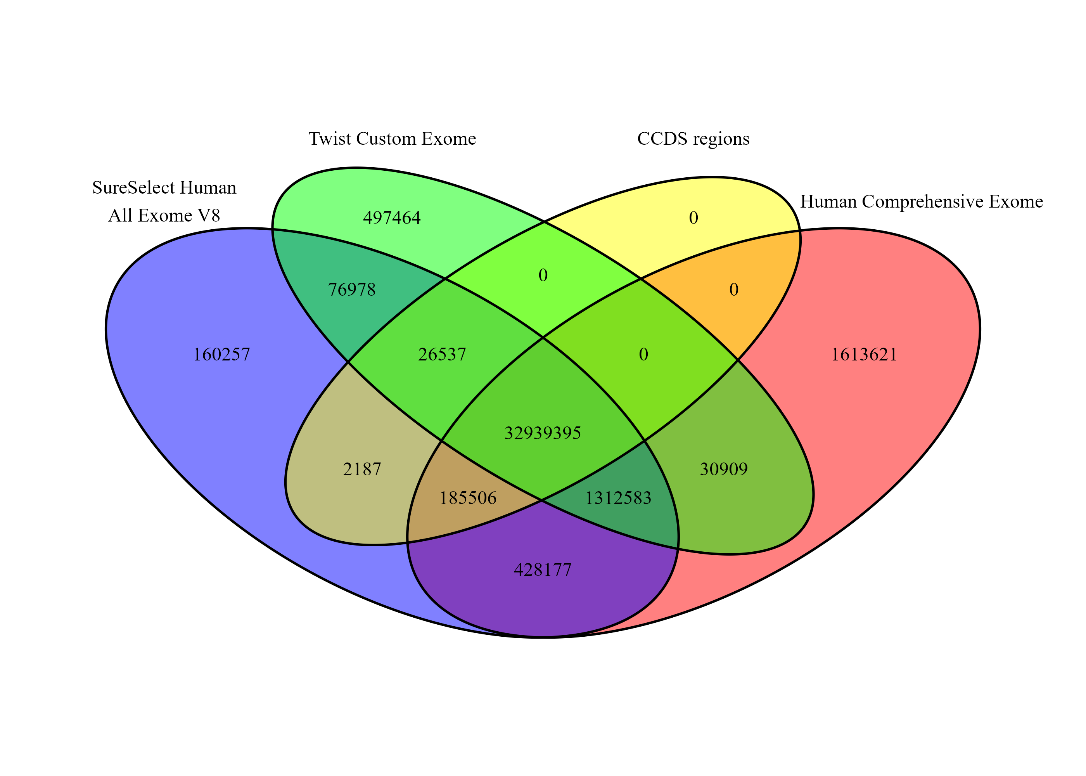
Supplementary Figure 1. Intersection of the targets of Agilent SureSelect Human All Exon V8, Twist Human Comprehensive Exome and Twist Custom Exome probes with CCDS regions. These 3 kits have the highest theoretical performance in capturing CCDS regions. Genomic region sizes in base pairs.

Supplementary Figure 2. Intersection of the targets of MGI Easy Exome Capture V5, Roche KAPA HyperExome V1 and MedGenome ExomeMax V2 probes with CCDS regions ± 25 bp padding. These 3 kits have the highest theoretical performance in capturing CCDS regions ± 25 bp padding. Genomic region sizes in base pairs.


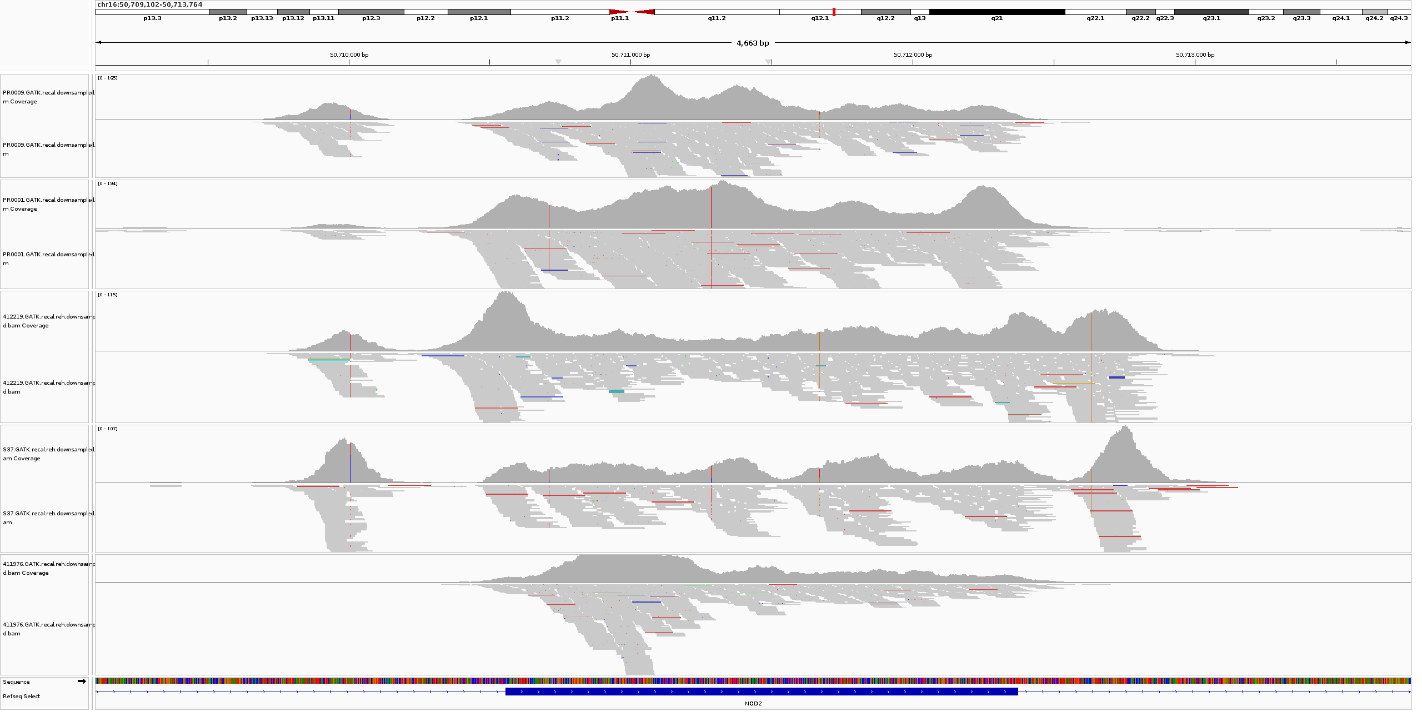
A)

B)


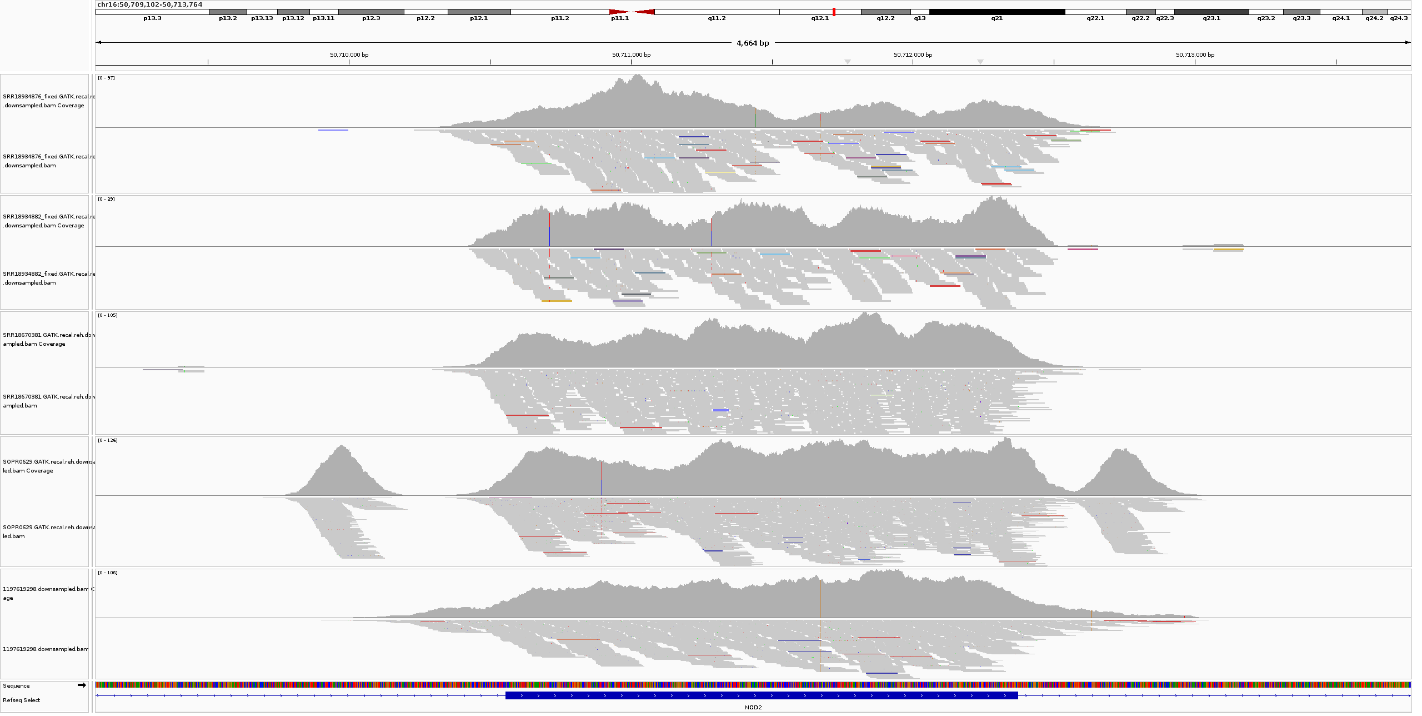


C)


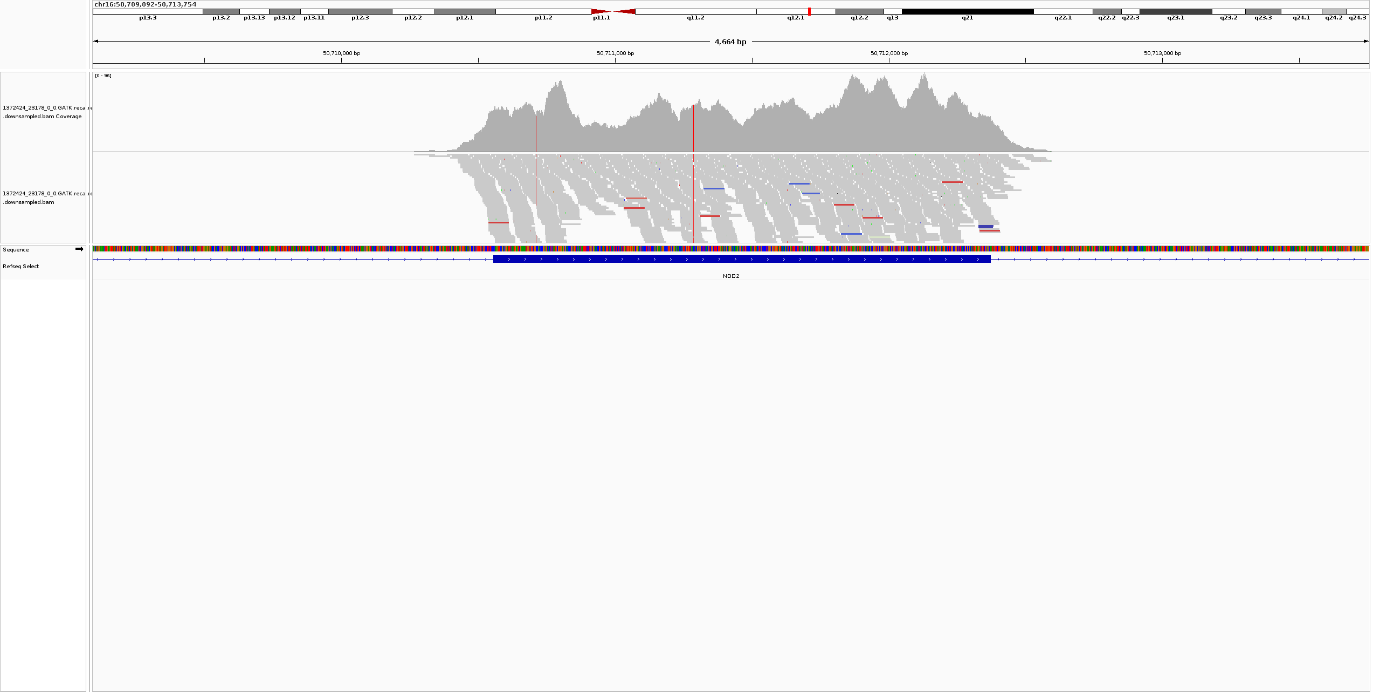


Supplementary Figure 3. IGV view of the coverage of exon 4 in the *NOD2* gene by one sample from each WES capture kit. All BAM files analysed were downsampled to 40,000,000 reads. A) Agilent SureSelect Human All Exon V5 and V6, MedGenome ExomeMax V2, Illumina DNA Prep with Exome 2.5 Enrichment, and MGI Easy Exome Capture V5. B) Agilent SureSelect Human All Exon V7 and V8, Roche KAPA HyperExome V1, Twist Human Comprehensive Exome, and Twist Custom Exome Capture. C) IDT xGen Exome Hybridisation Panel V1.
