# Supplementary material for: A systematic analysis of contemporary whole exome sequencing capture kits to optimise high-coverage capture of CCDS regions
Source: NAR Genom Bioinform. 2025 Sep 1;7(3):lqaf115. doi: 10.1093/nargab/lqaf115 (PMC12408908; doi:10.1093/nargab/lqaf115)
Supplement: lqaf115_Supplemental_Files [file lqaf115_supplemental_files.zip › Supplementary_Methods_20062025.docx]

**A Systematic Analysis of Contemporary Whole Exome Sequencing Capture Kits to Optimise High-Coverage Capture of CCDS Regions**

Fernando Vázquez López^a^, James J. Ashton PhD^ab^, Guo Cheng, PhD^ac^, Sarah Ennis, PhD^a^

^a^ Department of Human Genetics and Genomic Medicine, University of Southampton, Southampton, SO16 6YD, UK
^b^ Department of Paediatric Gastroenterology, Southampton Children’s Hospital, Southampton, SO16 6YD, UK
^c^ NIHR Southampton Biomedical Research Centre, University Hospital Southampton, Southampton, SO16 6YD, UK

**Supplementary Methods:**

Library Preparation and Sequencing for MGI Easy Exome Capture V5 and MedGenome ExomeMax V2 kits

Blood samples for the MGI Easy Exome Capture V5 kit were taken during the recruitment visit by venepuncture with a BD Vacutainer system, using whole blood/plasma evacuated tubes (BD Vacutainer® Plus K2EDTA Tubes) and sent to GenCell Pharma (Bogotá, Colombia), for genomic DNA extraction, NGS library preparation and sequencing. Extraction was performed using an automated magnetic bead purification method and an EXM3000 Nucleic Acid Isolation System (Zybio®). Library preparation was done with MGI Easy Exome Capture V5 Probe Set, which is designed to enrich exons in human protein-encoding genes, short flanking intronic regions, miRNA genes and mtDNA. Next generation paired-end sequencing was carried out using DNBSEQ Technology in a DNBSEQ-G400 sequencer (MGI®), which is based on the generation of DNA nanoballs (DNBs) for each DNA fragment created in the library and binding of each DNB on a patterned silicon chip (<https://en.mgi-tech.com/products/resources>).

For the MedGenome ExomeMax V2 kit, Genomic DNA was isolated from whole blood using the QIAamp DNA Blood Mini Kit (Qiagen, Germany) and quantified with a Qubit fluorometer (Thermo Fisher Scientific, USA). ~200 ng of DNA was used for library preparation. DNA was enzymatically fragmented to ~250 bp, end-repaired, 3′ adenylated, and ligated to indexed adapters. Adapter-ligated fragments were amplified using adapter-specific primers, followed by size selection and purification to generate the gDNA library. The library was hybridized and enriched with custom ExomeMax probes, then assessed for fragment size distribution via TapeStation (Agilent) and quantified using Qubit (Thermo Fisher Scientific) prior to sequencing.

Picard CollectHsMetrics

From the output of Picard CollectHsMetrics fields were extracted for: the percentage of aligned, unique, on-target bases (PCT_USABLE_BASES_ON_TARGET), the number of pass-filter reads (PF_READS), the number of unique pass-filter reads (PF_UNIQUE_READS), the number of unique pass-filter aligned reads (PF_UQ_READS_ALIGNED), the percentage of target bases captured at a coverage of 10X (PCT_TARGET_BASES_10X), the percentage of target bases captured at a coverage of 20X (PCT_TARGET_BASES_20X), the mean coverage depth across the target (MEAN_TARGET_COVERAGE), and the fold-80 base penalty (FOLD_80_BASE_PENALTY). Uniformity of coverage is reported as the Fold-80 Base Penalty, which reports the fold over-coverage (extra sequencing) necessary to raise 80% of target bases to the mean coverage level. The number of pass-filter reads was used to infer the corresponding number of bases based on read length. This was then used to calculate the number of aligned, unique, on-target bases and the number of pass-filter, aligned, unique, on-target reads.

Picard CollectInsertSizeMetrics

From the output of Picard CollectInsertSizeMetrics fields were extracted for: the median insert size of all paired end reads (MEDIAN_INSERT_SIZE), and the mean insert size (MEAN_INSERT_SIZE).
